# Editorial: Crosstalk between adipose tissue and immune cells

**DOI:** 10.3389/fendo.2022.1036737

**Published:** 2022-09-21

**Authors:** Marcus M. Seldin, Kosaku Shinoda, Prashant Rajbhandari

**Affiliations:** ^1^ Department of Biological Chemistry, University of California, Irvine, Irvine, CA, United States; ^2^ Division of Endocrinology & Diabetes, Department of Medicine, Albert Einstein College of Medicine, Bronx, NY, United States; ^3^ Diabetes, Obesity, and Metabolism Institute, Icahn School of Medicine at Mount Sinai, New York, NY, United States

**Keywords:** adipose tissue, adipose thermogenesis, neuroimmune cells, immune cells, obesity and type 2 diabetes, adipose tissue depots, fat reducing agents, cytokines

Adipose tissue is a critical regulator of systemic lipid and energy metabolism, and studies have shown that the cellular composition of adipose tissue plays an important role in the tissue’s ability to function as a metabolic equilibrium hub. Adipose tissue is composed of diverse cell types and is comprised of adipocytes and stromal vascular cells, containing mesenchymal stem cells, preadipocytes, fibroblasts, pericytes, smooth muscle cells, endothelial cells, and immune cells. Importantly, various immune cell types have been implicated in several different aspects of adipose tissue biology, including tissue homeostasis, thermogenesis, and regulation of adipose tissue function. Discovering and understanding the role of adipose immune cells in different states, such as physiological processes or metabolic dysregulation, will lead to a greater comprehension of the full breadth of adipose-immune biology and the possibility of developing therapeutics for chronic metabolic conditions, including obesity and diabetes.

Adipose tissue hosts a diverse population of immune cells that includes T cells, B cells, macrophages, dendritic cells, neutrophil, and mast cells. Proper functioning of these immune cells are critical for adipose tissue function and dysfunction of immune cells in obesity can lead to inflammation and obesity-associated metabolic disease. Immune cells can also regulate obesity-related disorders by either facilitating or inhibiting adipose thermogenesis. Thermogenic adipose tissue such as the brown adipose tissue (BAT), dissipates energy as heat and has been ascribed to an anti-obesity function in mice and humans. A specialized type of adipocytes known as “beige adipocytes” in white adipose tissue function similar to brown adipocytes and numerous studies have pointed towards a protective role of beige adipocytes against obesity. The role of immune cells in thermogenesis is comprehensibly summarized in a review by Agueda-Oyarzabal et al. where they describe different immune cell population and how these cells interact with thermogenic adipocytes in the adipose tissue microenvironment. The authors detail the general roles and specific functions of macrophages, T cells, B cells, eosinophils, innate lymphoid cells (ILC), γδT cells, natural killer T (NKT) cells, monocytes, and mast cells in thermogenic adipose depots. The review also evaluates the crosstalk between immune cells and adipocytes that is essential to maintain thermogenic capacity of adipocytes.

Adipose tissue is densely innervated by peripheral nerves of the sympathetic nervous system (SNS). Upon activation of SNS such as cold exposure, peripheral nerves secrete norepinephrine that acts on adipocytes to activate thermogenesis and lipolysis. Immune cells in adipose tissues are also closely associated with nerves and are involved in degrading norepinephrine, thereby limiting adipocyte thermogenesis and lipolysis. The role of neuroimmune cells beyond catecholamine degradation is largely unknown and Blaszkiewicz et al. build upon their prior studies and report an original work to show that a subset of cold-recruited myeloid lineage monocyte macrophages (Ly6c+CCR2+Cx3cr1+), labeled as cold-induced neuroimmune cells (CINCs) play a nerve-supporting role in the adipose tissue. They hypothesize that cold stress recruitment of CINCs produce brain derived neurotrophic factor (BDNF) to regulate neve innervation and neuro-adipose regulation of energy expenditure. Using RNA-seq, advanced confocal imaging in adipose tissue, and animal models, they comprehensibly demonstrate CINCs essential role in nerve survival and plasticity. Overall, their data reveal a unique role of immune cells in the maintenance of adipose innervation and metabolic function.

Adipose tissues can be found at different anatomical locations. The distribution of adipose tissue is of great importance in regards to obesity related co-morbidities. Insulin resistance often occurs when fat accumulates in intra-abdominal depots and is associated with a constellation of risk factors, in what is known as the metabolic syndrome. Mast cells residing in intra-abdominal depots such as the omental depot are generally considered to have deleterious role in adipose tissue. But recent studies have shown an important role of mast cells in adipose thermogenesis, adipogenesis, release of growth factors and bioactive molecules. Original research by Lopez-Perez et al. show a phenotypic alteration of mast cells in omental white adipose tissue (o-WAT) of obese humans with type 2 diabetes (T2D). Using flow cytometry based approach, the authors simultaneously measure mast cell population and expression of biologically active mast cell surface receptors in the o-WATs and subcutaneous adipose tissues (s-WAT) of non-T2D, pre-T2D, and T2D obese patients. They report a significant decrease of mast cell receptors expression in the o-WATs of T2D patients, implicating a serious defect in mast cell function. This study suggest an important role of mast cells in o-WAT physiology and factors that lead to the defect in mast cell survival, activation, and secretory function could drive the pathophysiology of T2D. Depending on the location, excess adipose tissue can also lead to cosmetic and psychological burden. Particularly, deposition of excess fat in the submental region also known as “double chin” has spurred the use of fat reducing compounds for cosmetic purposes. A review article by Muskat et al. comprehensibly summarizes the biology and mechanism of the two fat reducing compounds, Deoxycholic Acid (DCA) and Phosphatidylcholine Acid (PDC). The authors detail induction of cell apoptosis, inflammation, and fibrosis by DCA and PDC. They further evaluate a role of these compounds on lipolysis and report that DCA does not induce lipolysis and PDC induces lipolysis through release of inflammatory cytokine, tumor necrosis factor-alpha (TNFα). Overall, different fat depots harbor diverse immune cells types that have specialized role in regulating adipose tissue physiology and pathophysiology. A better understanding of the roles of tissue-resident or recruited immune cells in maintaining adipose tissue homeostasis could inform us about how obesity leads to a dysfunctional adipose microenvironment.

In conclusion, this Research Topic aims to incorporate reviews and novel data in the immunometabolism field related to the exciting relationship between adipose tissue and immune cells in different physiological and pathophysiologic contexts, with a focus on how the immune cells can control or interfere with whole-body metabolism.

## Author contributions

All authors listed have made a substantial, direct, and intellectual contribution to the work and approved it for publication.

## Acknowledgments

We would like to thank all the authors who have submitted manuscripts to this Research Topic. PR is supported by R00DK114571 and Diabetes Research and Education Foundation (DREF) Grant # 501. 

## Conflict of interest

The authors declare that the research was conducted in the absence of any commercial or financial relationships that could be construed as a potential conflict of interest.

## Publisher’s note

All claims expressed in this article are solely those of the authors and do not necessarily represent those of their affiliated organizations, or those of the publisher, the editors and the reviewers. Any product that may be evaluated in this article, or claim that may be made by its manufacturer, is not guaranteed or endorsed by the publisher.

